# Strong Association between Human and Animal *Brucella* Seropositivity in a Linked Study in Kenya, 2012–2013

**DOI:** 10.4269/ajtmh.15-0113

**Published:** 2015-08-05

**Authors:** Eric Mogaka Osoro, Peninah Munyua, Sylvia Omulo, Eric Ogola, Fredrick Ade, Peter Mbatha, Murithi Mbabu, Zipporah Ng'ang'a, Salome Kairu, Marybeth Maritim, Samuel M. Thumbi, Austine Bitek, Stella Gaichugi, Carol Rubin, Kariuki Njenga, Marta Guerra

**Affiliations:** Department of Preventive and Promotive Health, Ministry of Health, Nairobi, Kenya; Division of Global Health Protection, Centers for Disease Control and Prevention-Kenya, Nairobi, Kenya; Paul G. Allen School for Global Animal Health, Washington State University, Pullman, Washington; Center for Global Health Research, Kenya Medical Research Institute, Nairobi, Kenya; Directorate of Veterinary Services, Ministry of Agriculture, Livestock and Fisheries, Nairobi, Kenya; College of Health Sciences, Jomo Kenyatta University of Agriculture and Technology, Nairobi, Kenya; College of Health Sciences, University of Nairobi, Nairobi, Kenya; Bacterial Special Pathogens Branch, US Centers for Disease Control and Prevention, Atlanta, Georgia

## Abstract

Brucellosis is a common bacterial zoonotic infection but data on the prevalence among humans and animals is limited in Kenya. A cross-sectional survey was conducted in three counties practicing different livestock production systems to simultaneously assess the seroprevalence of, and risk factors for brucellosis among humans and their livestock (cattle, sheep, camels, and goats). A two-stage cluster sampling method with random selection of sublocations and households was conducted. Blood samples were collected from humans and animals and tested for *Brucella* immunoglobulin G (IgG) antibodies. Human and animal individual seroprevalence was 16% and 8%, respectively. Household and herd seroprevalence ranged from 5% to 73% and 6% to 68%, respectively. There was a 6-fold odds of human seropositivity in households with a seropositive animal compared with those without. Risk factors for human seropositivity included regular ingestion of raw milk (adjusted odds ratio [aOR] = 3.5, 95% confidence interval [CI] = 2.8–4.4), exposure to goats (herding, milking, and feeding) (aOR = 3.1, 95% CI = 2.5–3.8), and handling of animal hides (aOR = 1.8, 95% CI = 1.5–2.2). Attaining at least high school education and above was a protective factor for human seropositivity (aOR = 0.3, 95% CI = 0.3–0.4). This linked study provides evidence of a strong association between human and animal seropositivity at the household level.

## Introduction

Brucellosis, an infection caused by gram-negative bacteria of the genus *Brucella*, is an economically important zoonotic disease of humans, domesticated animals, marine mammals, and wildlife. Six main species of *Brucella* are recognized, four of which are identified as pathogenic to man. *Brucella melitensis,* which predominantly affects goats and sheep, is the most common cause of human brucellosis, whereas *B*. *аbortus,* found mainly in cattle, buffalo, elk, yaks, and camels, is the second most common cause of human infection. *B*. *suis,* which infects domestic pigs and rodents, and *B*. *canis* in canines are increasing in importance as sources of human brucellosis.^1^–^3^ The Food and Agriculture Organization (FAO), the World Health Organization (WHO), and the World Organisation for Animal Health (OIE) consider brucellosis to be one of the most widespread zoonosis causing substantial morbidity in both livestock and human populations globally.^4^ *Brucella* is also a potential agent for bioterrorism because of its propensity for airborne transmission and induction of a chronic debilitating disease that requires combined and lengthy antibiotic therapies.^5^

Human brucellosis is mainly transmitted from animal reservoirs through consumption of unpasteurized dairy products and undercooked meat products, inhalation of contaminated dust and contact with infected animal body fluids or tissues.^6^,^7^ Airborne transmission of *Brucella* to humans has also been documented in clinical laboratories and abattoirs.^4^ Owing to its low infectious dose, brucellosis poses an occupational risk for farmers, veterinarians, abattoir workers, laboratory personnel, and others who work with animals and consume their products.^8^,^9^ Clinically, human brucellosis presents as a febrile disease with varied clinical manifestations that are dependent on the stage of disease or the organs and systems involved. This feature makes the disease an important differential diagnosis for other important febrile diseases including malaria, tuberculosis, and typhoid. Brucellosis is rarely fatal but can be severely debilitating and disabling with a tendency for chronicity and persistence. Acute brucellosis has been estimated to have disability comparable to that of acute malaria, whereas chronic, localized brucellosis has been estimated to have disability comparable to osteoarticular disease.^10^ Brucellosis requires prolonged treatment with a combination of antibiotics, and can lead to permanent and disabling sequelae with considerable financial and economic consequences.^8^,^11^

In animals, although specific *Brucella* species predominantly infect cattle, sheep, goats, and camels, some *Brucella* species are multi-host and it is common, for example, for the highly virulent *B*. *melitensis* and *B*. *suis* to become established in cattle. Transmission of *Brucella* in animals is mainly through animal contact with infected aborted material, ingestion of contaminated pastures or milk. Sexual transmission can occur through natural mating or artificial insemination. The economic burden arising from brucellosis in animals is associated with productivity losses (longer calving intervals, reduced growth, increased incidences of abortion, infertility, and calf mortality), and restrictions on the trade and export of animals and their products.^12^

The control of brucellosis has been achieved in many developed countries. However, for other parts of the world including Latin America, the Middle East, Spain, parts of Africa, and western Asia brucellosis remains an endemic disease that causes more than 500,000 human infections each year.^2^,^13^ Most data on brucellosis in Africa have been reported from north Africa, although these reports likely underestimate the actual incidence. In Kenya, data on human brucellosis are scarce even though it is considered a priority disease.^14^ A study on patients presenting at a district hospital with flu-like symptoms reported 13% prevalence of brucellosis.^15^ Other studies have documented brucellosis exposure in animals in Kenya at varying levels.^16^,^17^

Despite the public health importance of brucellosis, its incidence and prevalence in animals and humans, as well as its socioeconomic impact, remain poorly understood in Kenya. In addition, despite its zoonotic transmission, few studies have investigated the burden and risk factors for animal and human brucellosis simultaneously among people and livestock living together. Taking into consideration different animal production systems, our objective was to determine the seroprevalence of *Brucella* exposure through the measurement of immunoglobulin G (IgG) antibody levels, and the risk factors for brucellosis among human and animal populations from the same household.

## Materials and Methods

### Study design and sample size determination.

**Figure 1.**
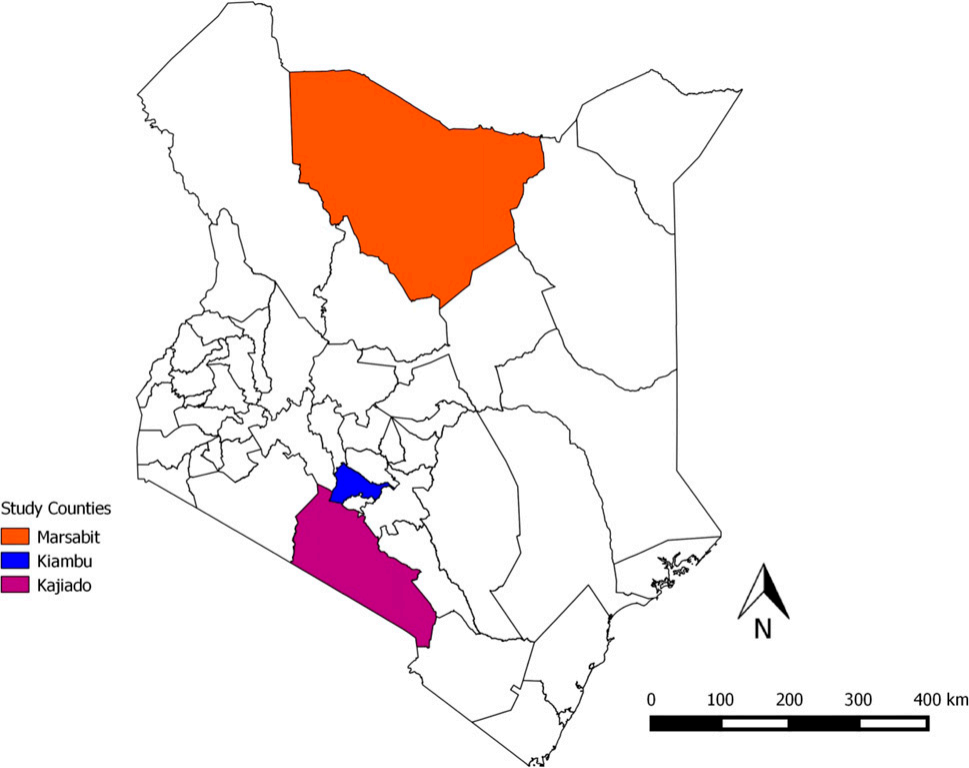
Map of Kenya showing the three counties where the study was carried out. Each study county represents a predominantly unique production system; Kiambu (small-holder system), Kajiado (agropastoral system) and Marsabit (pastoral system).^20^

We surveyed three administrative counties, each representing a different predominant livestock production system: Kiambu (small-holder system), Kajiado (agropastoral system) and Marsabit (pastoral system) (Table 1, Figure 1

).^18^–^20^ Administratively, each county is divided into subcounties, wards, and sublocations. Routine vaccination of animals against brucellosis is rare in all three counties. A cross-sectional study design was used. If a household consented to be part of the study, all persons over 5 years of age and livestock (cattle, sheep, goats and camels) living in the household were eligible for inclusion into the study.

We calculated the number of persons to be sampled based on an estimated seroprevalence of 50% for Kajiado and Marsabit and 5% for Kiambu, with an error margin of 5% and 2%, respectively, at the 95% confidence level.^21^ A design effect of two was applied to account for clustering and the sample size increased by 10% to account for nonresponse, resulting in 730 persons for Kajiado and Marsabit and 866 for Kiambu.

For animals, we used a seroprevalence of 15% for Kajiado and Marsabit and 2% in Kiambu^16^ with an absolute precision of 5% and 2%, respectively, at the 95% confidence level. To account for clustering, a design effect of seven was applied and the sample size increased by 10% to account for nonresponse. The sample size was estimated to be 1,605 animals per species in Kajiado and Marsabit and 1,548 per animal species in Kiambu.

### Sampling and data collection.

We applied a two-stage random sampling method to identify study households in each county. In the first stage, sublocations were stratified by predominant production system and a 10% random sample was subsequently selected in each stratum. This resulted in 21, 13, and 10 sublocations sampled in Kiambu, Kajiado, and Marsabit counties, respectively. In the second stage, we first calculated the number of households to be sampled within each sublocation based on the total human population in the sublocation, then randomly generated geographical coordinates using ArcGIS to correspond to the number of households to be sampled. Coordinates were loaded into a global positioning system device to guide study teams in identifying households. Each study team comprised a nurse, two veterinarians/paravets, a laboratory technologist, one/two animal handlers, and a village guide. Within the prescribed geocode, households were randomly identified using the “spin the bottle” method.^22^

A household was defined as a group of people who use a common cooking area. For each household, up to three persons aged 5 years and above were randomly selected and sampled. Sampling of the livestock was conducted per species (cattle, goat, sheep, and camels) in proportion to size of the herd. All animals in households with ≤ 15 animals were sampled while for farms with larger herd sizes, a maximum of 15 animals per species were sampled. Random animal selection was attempted in this case.

In Kiambu and Kajiado counties, blood samples were collected from eligible persons and from livestock between November and December 2012. Sampling in Marsabit took place in September 2013. Serum was separated from clotted blood by centrifugation at 3,000 × *g* for 15 minutes at designated health facilities. Two milliliters of serum were stored at −20°C for testing at the Center for Global Health Research, Kenya Medical Research Institute (KEMRI)/Centers for Disease Control and Prevention (CDC) laboratory in Kisumu (human samples), and the Central Veterinary Laboratory in Nairobi (animal samples).

Structured questionnaires pre-loaded into smartphones were administered by the team nurse to collect both household-level and individual-level risk factors for brucellosis. The team interviewed heads of households on types of livestock kept, handling of livestock and their products, consumption of animal products, history of brucellosis in the household, level of education, socioeconomic status, demographic characteristics, and herd management.

### Serological testing.

Human samples were tested using the IBL-America IgG enzyme-linked immunosorbent assay (ELISA) kits (Minneapolis, MN), whereas animal samples were tested using Svanova Biotech AB (Uppsala, Sweden) ELISA kits, that is SVANOVIR^®^ *Brucella*-Ab I-ELISA kit for cattle sera and SVANOVIR *Brucella*-Ab C-ELISA kit for camel, goat, and sheep sera. All assays were performed as recommended by the manufacturer. Briefly, human sera were diluted at 1:10 with sample diluent, added to microtitre plates pre-coated with *Brucella* antigen (*Brucella abortus*, strain W99; lysate of a NaCl extract) and incubated at room temperature for 1 hour. Conjugate was added and incubated for 30 minutes before adding substrate. The conjugate–substrate reaction was terminated after 20 minutes by adding a stop solution. Sample optical densities (ODs) were read at 450 nm. Both conjugate and substrate addition steps were preceded by a wash step, and all sera and controls were run in duplicates. Samples were interpreted as positive, equivocal, or negative based on readings of < 8, 8–12, and > 12, respectively, derived from a plot of the OD of standards (*y* axis, linear) and their concentrations (*x* axis, logarithmic). Equivocal samples were not included in subsequent analysis.

Cattle sera were first incubated in *Brucella* antigen-coated microtitre wells for 1 hour at 37°C followed by a wash step and similar incubation conditions with a conjugate. Substrate was then added following a wash step and the plates incubated at room temperature for 10 minutes before the reaction was terminated. Sample ODs were read at 450 nm and interpreted as positive or negative based on percent positivity (PP) cutoff values of < 40 or ≥ 40, respectively. All other animal sera were tested using competitive ELISA procedure. For this procedure, sera together with a mouse monoclonal antibody were exposed to *Brucella* smooth lipopolysaccharide (SLPS)-coated microtiter wells and incubated for 30 minutes, followed by a 30 minutes conjugate- and 20 minutes substrate incubation steps (all at room temperature) interspersed with wash steps. The reaction was stopped and ODs read at 450 nm. Percent inhibition (PI) values were then calculated and samples determined as negative for PI values < 30% or positive if ≥ 30%.

### Data analysis.

All analyses were done using STATA 12 (Stata Corporation, College Station, TX). Risk factor analysis was performed at the human, herd, and household levels, whereas univariate analysis was performed on human demographic factors (age, gender, education, and primary occupation). The animal-related human factors analyzed included livestock ownership, contact with or consumption of animals or their products, and development of disease symptoms within the last year. Factors characterizing animals or herds, such as breed, age, herd size, grazing system, and breeding system were also analyzed.

*Brucella* IgG serological status was used to indicate recent or past exposure to brucellosis. Seroprevalence was determined at individual, household, and herd level, and a seropositive household or herd was defined as any household with at least one seropositive human or animal, respectively. Individual seroprevalence was defined as the proportion positive for *Brucella* IgG among the sampled humans or animals.

Multivariate logistic regression was used to identify factors associated with brucellosis seropositivity and to estimate the magnitude of the adjusted odds ratios (aORs) for each factor while controlling for other confounding factors. To account for clustering, the svyset command in Stata 12 was used to specify clustering at sublocation and household levels. All analyses were subsequently carried out while accounting for clustering by applying the prefix svy in Stata 12. Univariate analysis was conducted for explanatory variables (biologically plausibly associated with brucellosis seropositivity) and those with a *P* value ≤ 0.2 were taken into the multivariate logistic regression model. In the multivariate model, backward stepwise selection was used until a minimum model in which all explanatory variables had *P* values < 0.05 were realized. Each dropped variable was added to the model separately to determine if it improved the fit. To determine the relationship between human brucellosis and animal brucellosis, data were summarized at the household level and the odds of human seropositivity were determined depending on herd seropositivity. Confidence intervals at 95% were determined and a *P* value < 0.05 was considered significant.

### Ethical considerations.

Ethical clearance and approval was obtained from the KEMRI Ethical Review Committee (ERC) and Animal Care and Use Committee (ACUC), and CDC Institutional review board. Other approvals were obtained from the Ministry of Health and the Ministry of Agriculture Livestock and Fisheries. Appropriate consenting processes were followed within households before sampling was initiated and data confidentiality was strictly maintained.

## Results

### Household characteristics.

A total of 1,088 households were surveyed; 505 (46%), 306 (28%), and 277 (26%) from Kiambu, Kajiado, and Marsabit counties, respectively. The mean household size was four persons (mean range = 4.1–4.5) in the three counties. Among household heads (HHD), 72% were males and the mean age was 52.0 years (range = 18–94 years). Five percent of the household heads in Marsabit County had at least high school education compared with 46% and 56% in Kajiado and Kiambu, respectively. Seventy-seven percent of the households owned at least one livestock species (sheep, goat, cattle, and camels). Among the livestock owning households, 73% owned cattle, 56% owned goats, 45% owned sheep, and 12% owned camels.

### Individual characteristics.

A total of 2,811 persons, 1,255 (45%) from Kiambu, 791 (28%) from Kajiado and 765 (27%) from Marsabit, consented and were interviewed. Fifty-four percent of the persons interviewed were female. The mean age was 35.6 years (range = 5–96 years). Table 2 below summarizes the sociodemographic variables of the study participants by county. A total of 11,028 livestock (37% goats, 28% sheep, 27% cattle, and 8% camels) were sampled. Camels were only sampled in Marsabit and comprised 16% of the livestock sampled in that county. Seventy-six percent of all sampled livestock were female whereas 27% of all sampled animals were sexually immature.

### Brucellosis seroprevalence.

#### Household and herd seroprevalence.

The highest household and herd prevalence was in Marsabit and the lowest in Kiambu (Table 3). Forty-two percent of all seropositive households had more than one seropositive individual. In Marsabit, 18% of the seropositive households had all three individuals sampled positive for *Brucella* IgG, whereas 70% of the seropositive herds had more than one animal seropositive compared with 27% and 18% of seropositive herds in Kajiado and Kiambu, respectively.

### Individual seroprevalence.

Human seroprevalence was generally 2- to 4-folds higher than animal seroprevalence in all the counties. Human and animal seroprevalences were highest in Marsabit County (Table 3). Human seroprevalence was three times higher in Marsabit compared with Kajiado and was six times higher in Kajiado compared with Kiambu. Similarly in livestock, seroprevalence was four times higher in Marsabit compared with Kajiado, which was about three times higher compared with Kiambu. Among the livestock species, camels and goats had the highest seroprevalence (Table 4). Seroprevalence among sexually immature animals was 3% compared with 10% among adult animals.

### Factors associated with herd and household seropositivity.

On univariate analysis, risk factors identified as significantly (*P* < 0.05) associated with human household seropositivity included: ownership of sheep, goats, or camels, pastoralist production system, nomadism, natural breeding (as opposed to artificial insemination), acquisition of new animals into farm, sold livestock from farm in previous 1 year, and male household head. The findings from the multivariate analysis are presented in Table 5. Factors associated with herd seropositivity by univariate analysis included rearing sheep, goats, or camels, pastoralist production system, nomadic movements, use of natural breeding, acquiring animals into farm, selling livestock from farm in previous 1 year, use of calving pens as opposed to open calving, and mingling with wildlife. The findings from the multivariate analysis are presented in Table 5.

### Human individual level factors.

On univariate analysis, significant factors (*P* < 0.5) that were protective against human seropositivity included attaining at least secondary education, consumption of processed dairy products, and previous treatment of febrile illness. Risk factors for human seropositivity on univariate analysis included male sex, using milk from own cattle, regularly ingesting raw milk, exposure to livestock (herding, feeding, and milking), assisting animals in delivery, handling hides, working with manure, cleaning barns, and history of febrile illness. On multivariate analysis, having secondary education and above was associated with decreased risk of human brucellosis seropositivity. Exposure to goats (herding, feeding, and milking), handling hides, and regular use of raw milk were risk factors associated with human brucellosis seropositivity (Table 6).

### Linking human and animal brucellosis seropositivity.

Univariate analysis determining whether animal brucellosis status was associated with human brucellosis at household level was conducted, and results are shown in Table 7. In all three study sites, the risk of human brucellosis increased up to six times with animal brucellosis seropositivity in a household. The odds were greatest for households where camels were seropositive for brucellosis.

## Discussion

This study used a “One Health” approach to simultaneously investigate the prevalence of brucellosis in humans and livestock living in the same households. Although animals are the main reservoirs for human brucellosis, studies on brucellosis have mainly been conducted separately in humans and animals.^4^,^17^,^23^ The uniqueness of this study design was the simultaneous study of brucellosis in people and animals living in the same households, allowing for identification and quantification of risk factors for brucellosis transmission at the household level. The study also provides a more complete epidemiological picture and deepens the understanding of infection patterns at the animal–human interface.^24^ The approach has the added value of enhancing closer cooperation between human and animal health practitioners.

Our study found that the odds of human seropositivity were six times higher in households with a seropositive animal compared with those without. Similar findings were reported in a study in Kyrgyzstan.^25^ This association was highest if the seropositive livestock was a camel or a goat consistent with the hypothesis that human risk of brucellosis is a function of the herd prevalence and depends both on the livestock species reared and human-livestock contact.^17^,^26^,^27^ Conversely, it contrasts results from a study in Togo and Mongolia where no association was found between human and animal seropositivity.^24^,^28^ It is notable, however, that sampling in Togo was done at the village level as opposed to household level. The study in Mongolia found no correlation between cattle and human seropositivity and only found a significant correlation with sheep, but not for goats. In that study, sampling was done at village level and livestock and human samples were not necessarily collected from the same households.^24^ We think that the difference in sampling level could account for the differences observed with our study.

Household and herd seroprevalence ranged from 5% to 73% and 6% to 68%, respectively, in the three counties with seroprevalence highest in Marsabit. These results are similar to those from another study conducted in Kenya, which reported a cattle seroprevalence ranging from 2% (agricultural high potential area) to 15% (semiarid, pastoralist area).^16^ Similar differences based on predominant production system have also been reported in Ethiopia and Uganda.^29^–^31^ The livestock seroprevalence (13.5%) observed among pastoralist communities in our study was lower than the 23% and 35% reported among pastoralist communities in Jordan and Egypt, respectively.^32^,^33^ Similarly, a higher livestock seroprevalence (6.5%) was found in an agro-based community in Uganda^34^ compared with Kiambu (1.2%).

The high seroprevalence in humans and livestock could reflect the endemicity of brucellosis in some parts of Kenya. Differences in human seroprevalence between the counties are likely due to the predominant production systems. Practices that promote brucellosis transmission such as drinking raw milk, nomadic movements, and use of common grazing and drinking areas for livestock are more likely in the pastoralist communities than agro-based communities.^17^

In this study, the seroprevalence among livestock species was fairly similar within a county, though slightly higher in goats or sheep. This finding could suggest that animal husbandry practices applied in different production systems influence transmission of brucellosis in livestock. In addition, there is possible cross-transmission of multiple *Brucella* species among different livestock species at the household and community level. Studies from Togo and Chad have found low human and small ruminant seroprevalence compared with that in cattle.^28^,^35^ In the Togo study, cattle seroprevalence was 9%, and < 1% among humans, whereas *Brucella* antibodies were not detected in sheep or goats. In our study, the seroprevalence in humans, goats, or sheep was proportionately higher compared with the studies in Togo and Chad, consistent with observations that *B*. *melitensis* that predominantly affects sheep and goats is important in brucellosis transmission to humans.^2^ Alternatively, the predominant *Brucella* species in the two countries could be different from those in Kenya where our study shows seropositivity in all species of livestock. In a study conducted in central Kenya, both *B*. *arbotus* and *B*. *melitensis* were isolated from bovine milk and aborted fetus samples.^36^

The human individual prevalence of 16% is higher than reported in other community level studies in Egypt (2%),^37^ Chad (4%),^35^ and Kyrgyzstan (9%).^25^ Seroprevalence studies in high-risk populations reported 8% among abattoir workers in Nigeria,^38^ 22% among slaughter house workers in Pakistan,^39^ and 40% in high-risk groups in Libya.^37^ These findings indicate high seroprevalence in humans in Kenya in the general population. This varying prevalence between counties is likely driven by the seroprevalence in livestock species, which also varies between counties. In our study, goat had the highest prevalence in Kajiado and Marsabit Counties, whereas sheep had the highest prevalence in Kiambu County. It is also likely related to different practices among the pastoralist communities in Marsabit and Kajiado, which promote transmission of brucellosis.

At the herd level, independent risk factors included keeping goats, keeping sheep, and use of calving pens. Goats and sheep often feed near homesteads where abortions due to brucellosis are likely to happen and could likely be exposed to contaminated environment for longer periods compared with the cattle and camels, increasing the likelihood of infection. Alternatively, goat and sheep could be more susceptible to *Brucella*. Farmers who use calving pens may not dispose the products of conception following calving, resulting in concentration and contamination of the environment and transmission of brucellosis within the herds. However, in Kiambu County, use of calving pens was found to be protective. This could be due to different practices in this high-potential agricultural production system compared with the pastoral production system including proper disposal of placentae and separating animals in peuperium from the rest of the herd for a longer time.

At the individual human level, risk factors included increasing age by decade, being male, regularly ingesting raw milk, exposure to goats (herding, milking, and feeding), and handling animal hides. These findings are consistent with findings from other studies, which indicate that the risk of human brucellosis is related to transmission through direct contact with animals or their products or indirectly through consumption of their products.^24^,^40^,^41^ The age-related increase in seroprevalence is consistent with an endemic pattern of infection in humans. In an endemic situation (where infection is continuously present), persons are increasingly likely to have become exposed with time and hence show increasing positivity with age. Male household heads are likely to propagate some cultural practices, which promote *Brucella* transmission, for example, not boiling milk before drinking. On the other hand, female household heads are likely to adhere to safer practices because of more frequent exposure to health workers while attending antenatal care or child welfare clinics. Persons who handle hides often have direct contact with raw meat and carcasses of infected animals, and through which infection probably occurs through cuts and wounds to bare hands, or through splashing of infected blood or other fluid to the conjunctiva.

This study has several limitations. First, the exclusion of children < 5 years of age limits the generalizability of our data to the entire population. Second, our testing methods report apparent rather than true prevalence. However, given the validity of the assays used, this difference is likely minimal. The ELISA tests used detected only antibodies against IgG but not IgM. It is possible that some participants could have had acute infection and be IgM positive but IgG negative, with the potential effect that the prevalence stated could be an underestimate. However, a study on diagnostic methods for brucellosis where IgG and IgM antibodies were measured simultaneously found little difference in the assays.^42^,^43^ Third, our use of pan-*Brucella* test kits limited our ability to distinguish antibodies against different *Brucella s*pecies. Fourth, given the cross-sectional nature of this survey, we could not assess temporal variations in seroprevalence if these existed.

## Conclusion

Our study gives evidence of a strong association between human and animal seropositivity at household level. In particular, goat and camel seropositivity was strongly associated with human seropositivity. There was higher prevalence of brucellosis in human and livestock in the predominantly pastoralist communities. To estimate the burden of brucellosis and identify appropriate interventions, it will be necessary to conduct further research to estimate the incidence of brucellosis with molecular typing of *Brucella* strains circulating among humans and livestock. Confirmation of transmission chains by molecular analysis of strains isolated from humans and different livestock species is warranted to determine the dominant brucella species.^44^ A socioeconomic study would give a societal perspective burden of the disease and help determine control measures to be undertaken in different settings.

## Figures and Tables

**Table 1:** Characteristics of the three study counties, Kenya, 2012–3013

	Kajiado	Kiambu	Marsabit
Human population^18^	687,312	1,623,282	291,166
Human population density per km^2^,^18^	31	638	4
Livestock population and distribution^19^	584,044 51.9% cattle, 27.0% sheep, 21.2% goats, 0.02% camels	1,832,045 22.5% cattle, 39.2% sheep, 38.2% goats, 0.1% camels	2,731,407 15.5% cattle, 35.1% sheep, 41.9% goats, 7.4% camels
Average annual rainfall^18^	700 mm	1,000 mm	100 mm
Climate	Semiarid	Tropical wet	Semiarid

**Table 2:** Sociodemographic characteristics of study respondents, 2012–2013

Characteristic	Kajiado (*N* = 791), No. (%)	Kiambu (*N* = 1,255), No. (%)	Marsabit (*N* = 765), No. (%)
Sex			
Female	422 (53.4)	719 (57.3)	380 (49.7)
Male	369 (46.6)	536 (42.7)	385 (50.3)
Mean age (SD)	34.9 (18.5)	36.7 (19.2)	34.3 (19.9)
Education level			
No education	201 (25.4)	57 (4.5)	518 (67.7)
Primary	335 (42.4)	569 (45.3)	191 (25.0)
Secondary	168 (21.2)	474 (37.8)	35 (4.6)
Post-secondary	84 (10.6)	152 (12.1)	16 (2.1)
Other	3 (0.4)	3 (0.2)	5 (0.7)
Occupation			
Works on farm/farmer	342 (43.2)	618 (49.2)	386 (50.5)
Salaried, off farm, skilled	49 (6.2)	130 (10.4)	42 (5.5)
Housewife	128 (16.2)	110 (8.8)	56 (7.3)
Salaried, off farm, unskilled	18 (2.3)	103 (8.2)	89 (11.6)
Student	157 (19.9)	274 (21.8)	175 (22.9)
Other	97 (12.3)	20 (1.6)	17 (2.2)

SD = standard deviation.

**Table 3:** Seroprevalence of brucellosis at household and herd level, 2012–2013

	Household seroprevalence (95% CI)	Herd seroprevalence (95% CI)
All counties	28.0 (24.1–32.4)	29.9 (25.8–34.2)
Kajiado	28.6 (21.0–37.7)	30.3 (23.3–38.5)
Kiambu	5.0 (3.3–7.5)	5.6 (3.6–9.2)
Marsabit	73.4 (65.6–80.0)	68.0 (59.8–75.3)

CI = confidence interval.

**Table 4:** Seroprevalence of brucellosis in humans and livestock species by county, 2012–2013

Seroprevalence	All counties % (95% CI)	Kajiado% (95% CI)	Kiambu % (95% CI)	Marsabit % (95% CI)
Human	16.4 (13.5–19.6)	15.3 (10.5–21.8)	2.4 (1.9–3.0)	46.5 (39.0–54.1)
Livestock	8.0 (6.8–9.4)	3.3 (2.8–4.1)	1.2 (1.0–1.5)	13.5 (11.2–16.2)
Cattle	4.1 (3.4–4.8)	3.3 (3.0–3.5)	0.8 (0.5–1.1)	11.2 (9.2–13.7)
Goat	10.7 (9.3–12.3)	3.6 (2.7–4.7)	1.3 (1.0–1.8)	16.1 (13.9–18.5)
Sheep	7.3 (6.1–8.8)	3.4 (2.8–4.1)	2.4 (1.9–3.1)	11.9 (10.2–13.5)
Camel	11.1 (7.1–17.0)	–	–	11.1 (9.4–15.0)

CI = confidence interval.

**Table 5:** Multivariate logistic regression analysis of the factors associated with human household and herd seropositivity

	All counties		Kajiado		Marsabit		Kiambu	
	aOR (95% CI)	*P* value	aOR (95% CI)	*P* value	aOR (95% CI)	*P* value	aOR (95% CI)	*P* value
Human household positivity								
Pastoral production system	6.8 (5.3–9.0)	< 0.001	2.9 (2.1–4.0)	< 0.001	–	–	42.7 (21.1–86.5)	< 0.001
Nomadic movements	3.4 (2.6–4.3)	< 0.001	2.3 (1.7–3.2)	< 0.001	5.7 (4.2–7.7)	< 0.001	–	–
Male household head	3.4 (2.9–3.9)	< 0.001	4.5 (3.4–5.9)	0.005	2.5 (2.0–3.0)	< 0.001	3.0 (2.0–4.7)	< 0.001
Sold livestock from farm in previous 1 year	1.7 (1.5–2.0)	< 0.001	2.2 (0.9–5.1)	0.074	1.4 (1.0–2.1)	0.054	2.1 (1.4–3.3)	0.001
Keeping cattle	0.5 (0.4–0.7)	< 0.001	0.6 (0.4–1.0)	0.048	0.9 (0.6–1.3)	0.487	1.1 (0.3–4.0)	0.891
HHD with at least secondary education	0.4 (0.3–0.5)	< 0.001	0.4 (0.4–0.5)	< 0.001	0.4 (0.1–0.6)	0.001	0.5 (0.4–0.7)	0.001
Herd Seropositivity								
Pastoral production system	9.8 (5.7–17.0)	< 0.001	2.9 (1.1–8.0)	0.039	–	–	–	–
Keeping goats	2.1 (1.3–3.7)	0.011	1.8 (0.6–5.6)	0.274	1.3 (0.5–3.6)	0.607	3.1 (1.0–9.7)	0.048
Keeping sheep	2.6 (1.6–4.1)	< 0.001	2.7 (0.9–7.7)	0.066	4.0 (1.7–9.3)	0.005	3.5 (1.2–10.5)	0.027
Use of calving pens	2.0 (1.3–3.2)	0.005	4.4 (1.6–11.6)	0.007	1.5 (0.7–3.4)	0.246	0.2 (0.0–1.8)	0.045
Exposure to aborted game	0.3 (0.2–0.6)	< 0.001	0.5 (0.2–1.2)	0.007	0.5 (0.2–1.8)	0.268	–	–

aOR = adjusted odds ratio; CI = confidence interval.

**Table 6:** Significant risk factors associated with human brucellosis exposure analyzed by multivariate logistic regression

	Combined		Kajiado		Kiambu		Marsabit	
	aOR (95% CI)	*P* value	aOR (95% CI)	*P* value	aOR (95% CI)	*P* value	aOR (95% CI)	*P* value
Age by decade	1.2 (1.1–1.2)	< 0.001	1.3 (1.2–1.4)	< 0.001	1.6 (1.5–1.6)	< 0.001	1.1 (1.0–1.2)	0.010
Male sex	1.6 (1.3–2.0)	< 0.001	1.0 (0.7–1.4)	0.832	1.3 (0.6–2.7)	0.479	3.0 (2.2–4.0)	< 0.001
Use of milk from own animals	2.6 (2.0–3.4)	< 0.001	2.0 (1.4–3.0)	0.001	1.3 (0.7–2.5)	0.410	3.2 (1.7–5.8)	0.002
Regular ingestion of raw milk	3.5 (2.8–4.4)	< 0.001	2.7 (1.9–3.9)	< 0.001	–	–	0.9 (0.6–1.4)	0.633
Assist in animal delivery	1.5 (1.2–2.0)	0.002	1.1 (0.6–2.0)	0.860	1.1 (0.7–11.7)	0.708	1.6 (1.1–2.3)	0.021
Exposure to sheep	1.6 (1.3–1.8)	< 0.001	3.2 (2.1–5.0)	< 0.001	0.6 (0.3–1.2)	0.135	2.0 (1.4–2.8)	0.002
Exposure to goats	3.1 (2.5–3.8)	< 0.001	1.5 (0.9–2.5)	0.127	0.9 (0.4–1.9)	0.792	2.1 (1.4–3.2)	0.004
Handling of animal hides	1.8 (1.5–2.2)	< 0.001	1.5 (1.2–2.0)	0.004	83.2 (24.9–278.7)	< 0.001	1.4 (1.1–1.8)	0.005
Secondary education and above	0.3 (0.3–0.4)	< 0.001	0.7 (0.5–0.9)	0.023	0.1 (0.0–0.5)	0.004	1.8 (0.4–7.7)	0.384

aOR = adjusted odds ratio; CI = confidence interval.

**Table 7:** Association between human and animal brucellosis seropositivity at household level

	OR	95% CI	*P* value
Livestock	6.2	5.5–7.1	< 0.001
Goats	10.7	9.0–12.8	< 0.001
Sheep	4.2	3.4–5.1	< 0.001
Cattle	2.7	2.1–3.4	< 0.001
Camel	11.0	8.3–14.7	< 0.001

CI = confidence interval; OR = odds ratio.
